# Acute Myeloid Leukemia Masquerading as Decompensated Cirrhosis

**DOI:** 10.7759/cureus.27538

**Published:** 2022-07-31

**Authors:** Hardeep S Ahdi, Seetharam Mannem, Asif Lakha

**Affiliations:** 1 Internal Medicine, Advocate Lutheran General Hospital, Park Ridge, USA; 2 Gastroenterology, Advocate Lutheran General Hospital, Park Ridge, USA

**Keywords:** decompensated liver cirrhosis, symptomatic anemia, decompensated cirrhosis, critical anemia, acute myeloid leukemia (aml)

## Abstract

Patients with known cirrhosis who present with anemia, thrombocytopenia, acute renal failure, and confusion are usually presenting with decompensated cirrhosis. We present a patient with known alcoholic cirrhosis presenting with the above abnormalities, initially thought to be decompensated cirrhosis but found to have acute myeloid leukemia (AML) with acute blast crisis. This case was presented as a poster at the American College of Gastroenterology Annual Scientific Meeting held on October 22-27, 2021.

A 59-year-old male with a history of compensated alcoholic cirrhosis presented with unresponsiveness. On physical exam, vitals were normal, he appeared lethargic with generalized pallor, and rectal exam demonstrated an empty rectal vault with no blood or stool noted. Labs were notable for hemoglobin 3.1 g/dL, platelet count 41,000/µL, creatinine 5.2mg/dL, aspartate aminotransferase (AST) 242 U/L, alanine aminotransferase (ALT) 138 U/L, bilirubin 0.8 mg/dL, lactic acid 8.5 mmol/L, international normalized ratio (INR) 1.8, ammonia 51µmol/L. Imaging with CT head was unremarkable and CT abdomen demonstrated cirrhotic morphology of the liver with a small amount of ascites. Upper endoscopy was performed with no evidence of varices. Paracentesis demonstrated a high serum-ascites albumin gradient with low total protein consistent with portal hypertension. He was intubated for airway protection due to worsening encephalopathy. A peripheral smear was performed which showed myeloblasts with no signs of hemolysis. Bone marrow biopsy was subsequently performed which revealed 38% myeloblasts and features of myelodysplastic syndrome suggestive of secondary AML. Chemotherapy was not initiated as he was acutely critically ill and he expired shortly thereafter.

AML can present with symptomatic anemia, bleeding, mental status changes due to central nervous system involvement, organomegaly, and renal insufficiency. Diagnosing AML in the setting of decompensated liver cirrhosis can be difficult as the clinical presentations can be similar at times. Thus, hematological causes should be considered when there is profound anemia with no acute blood loss early in the course.

## Introduction

Acute myeloid leukemia (AML) can present with a variety of different clinical manifestations, including anemia, thrombocytopenia, renal failure, and organomegaly. Diagnosing AML, especially in the setting of decompensated cirrhosis can be challenging as the presentation is similar. Anemia is the most common complication of liver cirrhosis and is seen in 75% of cases, as the etiology is often diverse and multifactorial, including but not limited to acute/chronic blood loss from suspected upper gastrointestinal (GI) bleeding, malnutrition, hypersplenism secondary to portal hypertension, and impaired coagulation [1]. Here, we present a patient with known alcoholic cirrhosis presenting with profound anemia, who was found to have AML. This case was presented as a poster at the American College of Gastroenterology Annual Scientific Meeting held on October 22-27, 2021.

## Case presentation

A 59-year-old male with a history of alcoholic cirrhosis (Model for End-Stage Liver Disease {MELD} Score 29 on admission) with no prior esophagogastroduodenoscopy (EGD), who initially presented to the emergency department for unresponsiveness, at which point was found to have profound anemia with hemoglobin 3.1g/dL and platelet count 41K/mcL. Upon arrival, vital signs were unremarkable. On exam, the patient appeared lethargic and pale, noted to have abdominal distension with a positive fluid wave without any signs of rebound tenderness, rigidity, guarding, bright right blood per digital rectal exam nor any active signs of bleeding. Labs were notable for white blood cell count 6.1 x10^9/L (differential includes: 62% neutrophils, 25% lymphocytes, 9% monoblasts, and 2% blasts), mean corpuscular volume (MCV) 137 fL, creatinine 5.2mg/dL (baseline 1.37mg/dL), elevated alanine transaminase (AST) 242 U/L, alanine aminotransferase (ALT) 138 U/L (baseline AST 57 U/L, ALT 30 U/L), lactic acidosis 8.5mmol/L, lipase 358U/L, international normalized ratio (INR) 1.8, Ammonia 51µmol/L, and lactate dehydrogenase (LDH) 665 U/L. The alcohol screen and drug screen were negative. CT head was negative. CT abdomen revealed nodular liver contour consistent with cirrhosis and a small amount of perihepatic ascitic fluid. The patient received four units of red blood cell transfusions with an appropriate rise in hemoglobin but no change in mental status.

**Figure 1:**
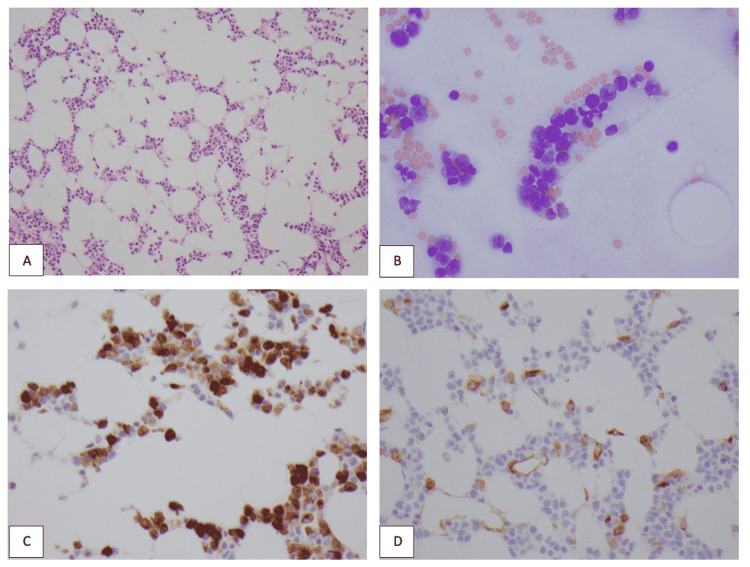
Histopathology A & B:  Aspirate smear and core biopsy show numerous myeloblasts and monoblasts C: CD34 immunohistochemical (IHC) highlights scattered myeloblasts D: Lysozyme IHC highlights monocytic cells including monoblasts

Esophagogastroduodenoscopy (EGD) was non-suggestive of varices or bleeding ulcers. He had diagnostic and therapeutic paracentesis with high serum ascites albumin gradient (SAAG) suggestive of portal hypertension. The patient was intubated for airway protection due to worsening encephalopathy. A peripheral smear was obtained given concern for thrombotic thrombocytopenic purpura (TTP) as lab results revealed severe anemia, acute renal failure, thrombocytopenia, and hemolysis (high LDH and direct antiglobulin test {DAT 1+} positive). However, the smear did not show signs of hemolysis nor revealed any schistocytes to support evidence for TTP. Instead, rare blasts were noted along with their corresponding immunohistochemistry staining, as seen in Figures 1A-1D. Bone marrow biopsy was subsequently performed which revealed AML with 38.5% blasts and myelodysplastic syndrome (MDS) features (secondary AML). Flow cytometry revealed an abnormal population of cells (9% of total cellular events) positive for cluster of differentiation (CD)13, CD15, CD33, CD34, CD45, and CD117 markers. Unfortunately, the patient was critically ill to even consider starting chemotherapy treatment. The patient rapidly decompensated and passed away.

## Discussion

Acute myeloid leukemia usually presents with symptomatic anemia, bleeding, mental status changes due to central nervous system involvement, organomegaly, and renal insufficiency. Diagnosis of AML in the setting of decompensated liver cirrhosis can be challenging as the presentation is similar. It should be considered when there is severe anemia and thrombocytopenia with no overt bleeding and multiorgan dysfunction. In our case, we noted severe anemia, thrombocytopenia, elevated transaminases, elevated lactic acid, acute kidney injury, and a small amount of perihepatic ascitic fluid on presentation which led us to exclude decompensated cirrhosis as the primary cause. Given this initial presentation, an EGD was performed to rule out esophageal variceal bleeding from decompensated cirrhosis. Hemolysis is usually not seen in cirrhosis but has been reported in AML [2]. We noticed DAT 1+ during the hemolysis screen which is insignificant given that haptoglobin and bilirubin were normal. In this patient, the profound anemia was thought to be multifactorial due to nutritional deficiency from alcoholic cirrhosis, chronic inflammation, renal disease, bone marrow failure due to hypo-proliferation, and hematological malignancies [3,4].

There were seven case reports described in the literature where AML presented as acute liver failure and obstructive jaundice. It is a rare presentation and gives rise to very high mortality [5]. Their presentation includes abnormal blood counts (anemia, leukocytosis, thrombocytopenia) and five of seven patients had blasts on peripheral smear, similar to our case. Myelocytes and blasts were revealed on a peripheral smear, with subsequent bone marrow biopsy confirming AML. When unexplained abnormalities are identified on peripheral blood smears in the setting of abnormal complete blood count (CBC) with or without physical exam findings (ie, hepatomegaly or splenomegaly), a bone marrow biopsy should be considered in order to quickly identify a diagnosis and initiate appropriate treatment therapy to provide the patient best chance at remission/survival.

Interestingly, our case differs from other published reports per literature review in that our patient eventually had a confirmed diagnosis of AML but without infiltrative liver involvement. It has been noted that hepatic involvement in AML has rarely been reported despite AML being one of the most common leukemias [6]. Other studies have shown acute liver failure in the setting of myelodysplastic syndrome (MDS) that transformed into AML with most cases presenting with cholestasis and obstructive jaundice, but few with transaminitis without evidence of cholestasis [6,7]. Similar to how others have described per the literature review, our patient was too ill to initiate treatment with chemotherapy and had an unfavorable outcome which unfortunately led to his demise from multiorgan failure.

## Conclusions

The presentation of AML is indeed multifaceted and has overlapping clinical presentations with decompensated cirrhosis due to similar presentation anemia, thrombocytopenia, encephalopathy, and renal failure. When patients with previously compensated cirrhosis present with the above, AML should be kept on the differential especially if workup does not suggest decompensated cirrhosis as the cause. Hematological causes should be considered when there is profound anemia with no acute blood loss early in the course as a timely definitive diagnosis (e.g., simple tests such as peripheral smear and bone marrow biopsy) could help with earlier onset of initiation of treatment.
